# In-silico activity prediction and docking studies of some flavonol derivatives as anti-prostate cancer agents based on Monte Carlo optimization

**DOI:** 10.1186/s13065-023-00999-y

**Published:** 2023-07-26

**Authors:** Faezeh Tajiani, Shahin Ahmadi, Shahram Lotfi, Parvin Kumar, Ali Almasirad

**Affiliations:** 1 Department of Medicinal Chemistry, Faculty of Pharmacy, Tehran Medical Sciences, Islamic Azad University, Tehran, Iran; 2 Department of Pharmaceutical Chemistry, Faculty of Pharmaceutical Chemistry, Tehran Medical Sciences, Islamic Azad University, Tehran, Iran; 3 Department of Chemistry, Payame Noor University (PNU), Tehran, 19395-4697 Iran; 4 Department of Chemistry, Kurukshetra University, Kurukshetra, Haryana 136119 India

**Keywords:** Flavonoids compounds, Prostate cancer, QSAR, CORAL software, Molecular docking

## Abstract

The QSAR models are employed to predict the anti-proliferative activity of 81 derivatives of flavonol against prostate cancer using the Monte Carlo algorithm based on the index of ideality of correlation (IIC) criterion. CORAL software is employed to design the QSAR models. The molecular structures of flavonols are demonstrated using the simplified molecular input line entry system (SMILES) notation. The models are developed with the hybrid optimal descriptors i.e. using both SMILES and hydrogen-suppressed molecular graph (HSG). The QSAR model developed for split 3 is selected as a prominent model (${R}_{Validation}^{2}$= 0.727, ${IIC}_{validation}$= 0.628, ${Q}_{Validation}^{2}$= 0.642, and ${\overline{r} }_{m}^{2}$=0.615). The model is interpreted mechanistically by identifying the characteristics responsible for the promoter of the increase or decrease. The structural attributes as promoters of increase of pIC_50_ were aliphatic carbon atom connected to double-bound (C…=…, aliphatic oxygen atom connected to aliphatic carbon (O…C…), branching on aromatic ring (c…(…), and aliphatic nitrogen (N…). The pIC_50_ of eight natural flavonols with pIC_50_ more than 4.0, were predicted by the best model. The molecular docking is also performed for natural flavonols on the PC-3 cell line using the protein (PDB: 3RUK).

**Supplementary Information:**

The online version contains supplementary material available at 10.1186/s13065-023-00999-y.

## Introduction

Flavonoids are a class of polyphenolic compounds which possess a phenyl benzopyrone structure (C6–C3–C6) and are present in all vascular plants. These are produced as secondary plant metabolites, which are known to demonstrate broad-spectrum pharmacological activities, but the human body is unable to produce them [1–3]. These compounds according to saturation level subdivided into flavanols, flavonols, flavones, flavanones, isoflavones, flavanonols, and chalcones [4, 5].

The CYP17A1 has an important role in the biosynthesis of dehydroepiandrosterone (DHEA) as the precursor of androgens and overexpression of this enzyme can cause prostate cancer. Abiraterone as an approved anti-prostate cancer drug is a CYP17A1 inhibitor [6, 7]. Flavonols are characterized by a hydroxyl group present at C-3 of the flavone skeleton and there are some reports about the CYP17A1 inhibitory activity of flavonoids like rutin, morusflavone, quercetin, kaempferol and isorhamnetin [8–10].

These have also been attracted by medicinal chemists because of their effective anti-prostate cancer properties. Prostate cancer is the most common type of diagnosed cancer among males worldwide with the incidence of 28 cases per 100,000 and mortality being 7 per 100,000 [11–13]. Normal growth and maintenance of the prostate is dependent on androgen hormones that act through the androgen receptor. Activation of the androgen receptor drives the development of prostate cancer. It has been reported that the agents such as flavonols that down-regulate androgen receptors can inhibit the development of prostate cancer cells [14–16].

The influence of chemical structures of flavonols over their anticancer activities has been investigated experimentally and shown that structural modification can further increase its anti-cancer activity and ability to activate PC-3 cell apoptosis. However, the structure–activity relationship for flavonols as anti-prostate cancer agents has captured attention by quantitatively correlating the molecular structures or properties with variation in pharmacological activity [17, 18].

The anti-prostate cancer activity is expressed typically with IC_50_ (half maximal inhibitory concentration) values. Quantitative structure–activity relationships (QSARs) are a powerful tool to predict IC_50_ of flavonoids in general. Already, no study has been reported on QSAR modeling for predicting the IC_50_ of flavonols against prostate cancer.

QSAR model is a mathematical equation which is widely employed to estimate and predict pharmacological activity or physical, chemical properties/activities of chemicals using descriptors derived from chemical structure [19–22]. The CORAL (Correlation and Logic) freeware software is employed for designing the Quantitative structure–activity/activity relationships (QSPRs/QSARs) models in compliance with OECD principles [23–26]. In CORAL software, the SMILES notations of the molecular structure are used as an input file and produce the best model based on Monte Carlo optimization [27–30]. It can be applied to compute the optimal descriptor by using solely SMILES or molecular graph-based descriptor or a combination of both descriptors (so-called hybrid descriptor). A literature survey reveals that the index of ideality of correlation (IIC) parameter of CORAL software can be employed to build robust QSAR models [31–34].

Molecular docking simulation is a computational methodology that purveys automatic tools to measure the conformation of a protein–ligand complex. The aim of molecular docking is to regulate the position of the ligand in the protein. An energy-based scoring function is commonly used in docking procedures to find the energetically most advantageous ligand conformation when attached to the target. Intermittently, the Monte Carlo computational methodologies are also applied in molecular docking simulation [35, 36].

Since ancient times various natural products have been used as traditional medicine against various human diseases. Moreover, natural products are easily applicable, cheap, accessible and acceptable treatment method with minimum cytotoxicity [37]. As a results of QSAR modeling, the pIC_50_ activity of some natural flavonols as anti-proliferative agents were predicted and reported.

The goal of this report is to devise reliable first QSAR models utilizing CORAL software to predict pIC_50_ of 81 flavonols against prostate cancer. In the development of QSAR models, a hybrid optimal descriptor, a combination of SMILES and hydrogen suppressed graph (HSG), is employed. The index of ideality of correlation (IIC) is used to improve the predictive potential of QSAR models. Further, the pIC_50_ is also calculated for a series of eight natural flavonols using the QSAR models of all splits. As mentioned above flavonols show their anti-prostate cancer activity through different mechanism of actions. However, molecular docking is also performed for eight natural flavonol derivatives in order to evaluate their potential affinity to CYP17A1 (PDB: 3RUK).

## Methods

### Data

Experimental data on anti-prostate cancer (PC-3) activities of 86 flavonols were taken from the four literature reports (Additional file 1: Table S1) [11, 38–40]. The numerical values of activity were converted to a negative logarithmic scale, pIC_50_ (− logIC_50_) (Molar) for QSAR modelling. The range of pIC_50_ for PC-3 cell line was from 3.39 to 6.28. The current dataset was not previously used for QSAR modeling. The molecular structures of the flavonol derivatives were sketched by BIOVIADraw 2019 and transferred to the SMILES code for modeling with the CORAL software. Three splits were made from the dataset and each split was further randomly divided into four sets i.e., training (≈ 35%), invisible training (≈ 25%), calibration (≈ 15%), and validation (≈ 25%) sets. In CORAL-based QSAR modeling, each set was assigned its specific accountability. The task of the training set (TRN) was to compute correlation weights and the task of the invisible training set (iTRN) was to control the adaptability of the data which were not employed in the training set. The assignment of the calibration set (CAL) was to detect the overtraining whereas the final estimation of the predictive potential of the designed QSAR model was assigned to the validation set (VAL) [34, 41].

### Hybrid optimal descriptor

Herrin, the optimal hybrid optimal descriptor based on SMILES and HSG was employed to create QSAR models for pIC_50_ of flavonol compounds. The literature reports showed that the QSPR models produced through the ‘hybrid’ optimal descriptor had better statistical parameters than the model designed by individually SMILES or HSG descriptors [42, 43].

The QSAR model employed to predict pIC_50_ of flavonol derivates is demonstrated in the following equation:

1 $${pIC}_{50}={\mathrm{C}}_{0}+{\mathrm{C}}_{1}\times {}^{Hybrid}\mathrm{DCW}\left({\mathrm{T}}^{*}, {\mathrm{N}}^{*}\right).$$

Here, C_0_ is the regression coefficient and C_1_ is the slope computed by the least-squares method; DCW (descriptor of correlation weights) is computed with correlation weights of molecular features extracted from HSG and SMILES notations. The following equation is employed to compute DCW:

2 $$DCW\left({T}^{*},{N}^{*}\right)=\sum CW({A}_{K}),$$

where A_K_ is an attribute of SMILES or HSG, the T* and N* define the threshold value and number of epochs of the Monte Carlo optimization, respectively.

3 $${}^{\mathrm{Hybrid}}\mathrm{DCW}\left({\mathrm{T}}^{*}, {\mathrm{N}}^{*}\right)={}^{\mathrm{SMILES}}\mathrm{DCW}\left(\mathrm{T}, {\mathrm{N}}^{*}\right)+{}^{\mathrm{Graph}}\mathrm{DCW}\left({\mathrm{T}}^{*}, {\mathrm{N}}^{*}\right).$$

The DCW of HSG and SMILES employed here are illustrated as Eqs. (4) and (5):

4 $$\begin{aligned}{}^{SMILS}\mathrm{DCW}\left(\mathrm{T},\mathrm{ N}\right)= &\sum \mathrm{CW}\left({\mathrm{S}}_{\mathrm{k}}\right) +\sum \mathrm{CW}\left({\mathrm{SS}}_{\mathrm{k}}\right)+\mathrm{CW}\left(\mathrm{BOND}\right)+\mathrm{CW}\left(\mathrm{NOSP}\right)+\mathrm{CW}\left(\mathrm{HARD}\right)+\mathrm{CW}\left(\mathrm{PAIR}\right)\\&+\,\mathrm{CW}\left(\mathrm{Cmax}\right)+\mathrm{CW}\left(\mathrm{Nmax}\right)+\mathrm{CW}\left(\mathrm{Omax}\right) \end{aligned}$$

5 $$\begin{aligned}{}^{HSG}\mathrm{DCW}\left(\mathrm{T},\mathrm{ N}\right)= & \sum \mathrm{CW}\left({\mathrm{e}1}_{\mathrm{k}}\right)+\sum \mathrm{CW}\left({\mathrm{e}2}_{\mathrm{k}}\right)+\sum \mathrm{CW}\left({\mathrm{e}1}_{\mathrm{k}}+{\mathrm{e}2}_{k}\right)+\sum \mathrm{CW}\left(\left|{\mathrm{e}1}_{\mathrm{k}}-{\mathrm{e}2}_{k}\right|\right)\\&+\sum \mathrm{CW}\left({\mathrm{pt}2}_{\mathrm{k}}\right)+\sum \mathrm{CW}\left({\mathrm{pt}3}_{\mathrm{k}}\right)\\&+\sum \mathrm{CW}\left({\mathrm{pt}2}_{\mathrm{k}}+{\mathrm{pt}3}_{\mathrm{k}}\right)+\sum \mathrm{CW}\left(\left|{\mathrm{pt}2}_{\mathrm{k}}-{\mathrm{pt}3}_{\mathrm{k}}\right|\right)+\sum \mathrm{CW}\left({\mathrm{S}2}_{\mathrm{k}}\right)+\sum \mathrm{CW}\left({\mathrm{S}3}_{\mathrm{k}}\right)+\sum \mathrm{CW}\left({\mathrm{S}2}_{\mathrm{k}}+{\mathrm{S}3}_{\mathrm{k}}\right)\\&+\sum \mathrm{CW}\left(\left|{\mathrm{S}2}_{\mathrm{k}}-\mathrm{S}3\mathrm{k}\right|\right)+\mathrm{CW}\left(\mathrm{C}5\right)+\mathrm{CW}\left(\mathrm{C}6\right)\end{aligned}$$

The SMILES attributes and HSG invariant applied in Eqs. (4) and (5) are depicted in Table 1.

**Table 1:** The detailed description of SMILES attributes and graph invariants for constructed models of pIC_50_

	ID	Definition
SMILES attribute	Sk	SMILES atom, *i.e.*, one symbol (e.g. ‘C’, ‘N’, ‘=’, etc.) or a group of symbols that cannot be examined separately (*e.g.*, ‘Cl’, ‘Br’, Si’, etc.)
	SSk	a mixture of two SMILES-atoms
	BOND	Presence or absence of chemical bonds: double (=), triple (#), and stereochemical (@) or @@)
	PAIR	Association two of BOND, NOSP, and HALO
	HARD	Association of BOND, NOSP, and HALO in the united structural code
	NOSP	Presence or absence of different chemical elements: nitrogen (N), oxygen (O), sulfur (S), and phosphorus (P);
	C_max_	Maximum number of rings
	N_max_	Maximum number of nitrogen atoms in a molecule
	O_max_	Maximum number of oxygen atoms in a molecule structure
Graph invariant	e2k	Morgan extended connectivity of first order
	e3k	Morgan extended connectivity of second-order
	pt2k	Number of paths of lengths 2 and 3 starting from a given vertex in the graph
	pt3k	Number of paths of length 3 starting from a given vertex in the graph
	S2_k_	Valence shells of the second orders
	S3_k_	Valence shells of the third orders
	C5 and C6	Codes of rings (five-member and six-member rings, with the data on the presence or absence of heteroatoms, aromaticity, and the total number of given rings in the molecule)

A flowchart of a Monte Carlo optimization cycle is presented by Sokolovic et al. [44]. At first cycle, the CW(x) of features is randomly generated and then optimized based on the proposed objective function. Herein, two kinds of target functions consisting of the balance of correlation without IIC (TF1) and the balance of correlation with IIC (TF2) are studied.

The following mathematical equation is employed to compute the TF_1_ and TF_2_:

6 $${TF}_{1}={R}_{TRN}+{R}_{iTRN}-\left|{R}_{TRN}-{R}_{iTRN}\right|\times Const$$

7 $${TF}_{2}={TF}_{1}+{IIC}_{CAL}\times Const$$

The R_training_ and R_invTraining_ are the correlation coefficients for the training and invisible training sets, respectively. The empirical constant (Const) is usually fixed [45, 46].

The IIC_CAL_ is calculated with data on the calibration (CAL) set as the following:

8 $$\mathrm{IIC}={\mathrm{R}}_{\mathrm{C}AL}\times \frac{\mathrm{min}({}^{-}{\mathrm{MAE}}_{\mathrm{CAL}}, {}^{+}{\mathrm{MAE}}_{\mathrm{CAL}})}{\mathrm{max}({}^{-}{\mathrm{MAE}}_{\mathrm{CAL}}, {}^{+}{\mathrm{MAE}}_{\mathrm{CAL}})}$$

R_CAL_ is the correlation coefficient for the calibration set. The negative and positive mean absolute errors are shown with ^−^MAE and ^+^MAE, which are computed using the following equations:

9 $${}^{-}{\mathrm{MAE}}_{\mathrm{CAL}}=-\frac{1}{\mathrm{N}}\sum_{y=1}^{{N}^{-}}\left|{\Delta }_{\mathrm{k}}\right| \quad {\Delta }_{\mathrm{k}} < 0, {}^{-}\mathrm{N\,is\,the\,number\,of\,}{\Delta }_{\mathrm{k}} < 0$$

10 $${}^{+}{\mathrm{MAE}}_{\mathrm{CAL}}=+\frac{1}{\mathrm{N}}\sum_{y=1}^{{N}^{+}}\left|{\Delta }_{\mathrm{k}}\right| \quad {\Delta }_{\mathrm{k}}\ge 0, {}^{+}\mathrm{N\,is\,the\,number\,of\,}{\Delta }_{\mathrm{k}}\ge 0$$

11 $${\Delta }_{\mathrm{k}}={\mathrm{Observed}}_{\mathrm{k}}-{\mathrm{Calculated}}_{\mathrm{k}}$$

The ‘k’ is the index (1, 2,…N). The observed_k_ and calculated_k_ are related to numerical values of the endpoint.

This IIC is obtained by using the correlation coefficient between the observed and predicted values of the endpoint for the calibration set, taking into account the positive and negative dispersions between the observed and calculated values [47].

### Applicability domain

The applicability domain (AD) is another key guideline that should be included in a built QSPR/QSAR model. It was defined by the OECD as "the response and chemical structure space in which the model produces predictions with a specified reliability" [48, 49]. The CORAL-based QSAR model computes AD based on the dispersion of SMILES features in the training and calibration sets [50]. The AD is defined as ‘DefectA_K_’, which was computed with the following equation:

12 $$\begin{aligned}&{\mathrm{Defect}}_{{\mathrm{A}}_{\mathrm{K}}}=\frac{\left|{\mathrm{P}}_{\mathrm{TRN}}{(\mathrm{A}}_{\mathrm{K}})-{\mathrm{P}}_{\mathrm{CAL}}{(\mathrm{A}}_{\mathrm{K}})\right|}{{\mathrm{N}}_{\mathrm{TRN}}{(\mathrm{A}}_{\mathrm{K}})+{\mathrm{N}}_{\mathrm{CAL}}{(\mathrm{A}}_{\mathrm{K}})} \quad \mathrm{ If\, }{\mathrm{A}}_{\mathrm{K}}>0\\&{\mathrm{Defect}}_{{\mathrm{A}}_{\mathrm{K}}}=1 \quad \mathrm{ If\,}{\mathrm{A}}_{\mathrm{K}}=0 \end{aligned}$$

${P}_{TRN}{(A}_{K})$ and ${P}_{CAL}{(A}_{K})$ are the probability of an attribute 'A_k_' in the training and the calibration sets; ${N}_{TRN}{(A}_{K})$ and ${N}_{CAL}{(A}_{K})$ are the number of times of A_k_ in the training and calibration sets, respectively.

The statistical defect is computed using the following equation:

13 $${\mathrm{Defect}}_{\mathrm{Molecule}}=\sum_{\mathrm{k}=1}^{N{\mathrm{A}}}{\mathrm{Defect}}_{{\mathrm{A}}_{\mathrm{K}}}$$

N_A_ is the number of active SMILES attributes for the given compounds.

In CORAL, a substance is an outlier if inequality 14 is fulfilled:

14 $${\mathrm{Defect}}_{\mathrm{molecule}} >2\times {\overline{\mathrm{Defect}} }_{\mathrm{TRN}}$$

${\overline{\mathrm{Defect}} }_{\mathrm{TRN}}$ is an average of statistical defect for the dataset of the training set.

### Validation of the model

It is most important to validate the predictive potential of a constructed QSAR model. In the present manuscript, the reliability and robustness of the QSAR models were verified using the following three methodologies: i) internal validation or cross-validation by considering the training dataset, ii) external validation by considering the prediction set and iii) data randomization or Y-scrambling.

The various standard statistical metrics such as correlation coefficient (R^2^), cross-validated correlation coefficient (Q^2^), concordance correlation coefficient (CCC), the IIC, ${Q}_{F1}^{2}$, ${Q}_{F2}^{2}$, and ${Q}_{F3}^{2}$, standard error of estimation (s), mean absolute error (MAE), Fischer ratio (F), novel metrics (${r}_{m}^{2}$) and Y-scrambling (${\mathrm{c}}_{{R}_{p}^{2}})$ were employed to validate the developed QSAR models. The mathematical equations of various validation metrics are shown in Table 2.

**Table 2:** The mathematical equation of different statistical benchmark of the predictive potential for CORAL models

Criterion of the predictive potential	Comments	Refs.
${R}^{2}=1-\frac{\sum {({Y}_{obs}-{Y}_{prd})}^{2}}{\sum {({Y}_{obs}-{\overline{Y} }_{train})}^{2}}$	Y_obs_ is the observed endpoint for the training set, and Y_pred_ is the predicted endpoint values for the training set of compounds ${\overline{Y}}_{train}$ is the mean observed endpoint of the training set	
${Q}^{2}=1-\frac{\sum {({Y}_{prd(train)}-{Y}_{obs(train)})}^{2}}{\sum {({Y}_{obs(train)}-{\overline{Y}}_{train})}^{2}}$	Y_obs(train)_ is the observed endpoint, and Y_pred(train)_ is the predicted response of the training set compounds	
$CCC=\frac{2\sum ({X}_{i}-\overline{X })({Y}_{i}-\overline{Y })}{\sum_{i=1}^{n}{({X}_{i}-\overline{X })}^{2}+\sum_{i=1}^{n}{({X}_{i}-\overline{X })}^{2}+n(\overline{X }-\overline{Y })}$	n is the number of compounds, and x_i_ and y_i_ denote the mean of observed and predicted values, respectively	[51]
${\mathrm{c}}_{{R}_{p}^{2}}=R\sqrt{\left({R}^{2}-{R}_{r}^{2}\right)}$	${R}^{2}$ is squared correlation coefficient of models and ${R}_{r}^{2}$ is squared mean correlation coefficient of randomized models	[52]
$\overline{{r }_{m}^{2}}=\frac{{r}_{m}^{2}+{r}_{m}^{{\prime}2}}{2}$	r^2^ is the squared correlation coefficient value between observed and predicted endpoint values, and ${\mathrm{r}}_{0}^{2}$ and ${\mathrm{r{\prime}}}_{0}^{2}$ are the respective squared correlation coefficients when the regression line is passed through the origin by interchanging the axes For the acceptable prediction, the value of all $\Delta {\mathrm{r}}_{\mathrm{m}}^{2}$ metrics should preferably be lower than 0.2 provided that the value of r^2^m is more than 0.5	[53]
$$\Delta {r}_{m}^{2}=\left|{r}_{m}^{2}-{r{\prime}}_{m}^{2}\right|$$		
$${r}_{m}^{2}={r}^{2}\times \left(1-\sqrt{{r}^{2}-{r}_{0}^{2}}\right)$$		
$${r{\prime}}_{m}^{2}={r}^{2}\times \left(1-\sqrt{{r}^{2}-{r{\prime}}_{0}^{2}}\right)$$		
$$MAE=\frac{1}{n}\times \sum \left|{Y}_{obs}-{Y}_{prd}\right|$$		

R^2^ statistic is a metric to evaluate the goodness of fit of a regression analysis. It measures the variation of experimental data with the predicted ones. The range of R^2^ is between 0 (no correlation) and 1 (perfect fit). R^2^ cross‐validated (Q^2^) is used for internal validation. The concordance correlation coefficient (CCC) is calculated to measure both precision and accuracy detecting how far each observation deviate from the best-fit. The CCC is calculated to detect both precision and accuracy distance of the observations from the fitting line and the degree of deviation of the regression line from that passing through the origin, respectively [51]. A lower value of MAE and s is desirable for good internal/external predictivity. Roy et al. [54] introduced a new metric ${\mathrm{r}}_{\mathrm{m}}^{2}$ that penalizes the r^2^ value of a model when there is large deviation between r2 and ${\mathrm{r}}_{0}^{2}$ values (Table 2). For a reliable QSAR model, the $\overline{{r }_{m}^{2}}$ and $\Delta {r}_{m}^{2}$ should be greater than 0.5 and smaller than 0.2, respectively. Y-scrambling or Y-randomization is an assessment to ensure the developed QSAR model is not due to chance, thereby giving an idea of model robustness [52]. For a robust QSAR model, Todeschini ${\mathrm{c}}_{{R}_{p}^{2}}$ parameter [55] is also calculated which should be more than 0.5. One of the important statistical parameters to judge different QSAR models is $\overline{{r }_{m}^{2}}$ for test set. Here, this parameter is used to select best model between six proposed models.

### Model interpretation

A straightforward process for the structural interpretation of QSPR/QSAR models is provided by the CORAL application. Three types of attributes may be identified by computing the correlation weights across several iterations of the Monte Carlo optimization algorithm. The positive numerical value of CWs in every iteration is considered for endpoint increase, the attributes with a negative value of CWs in every iteration is a notation for endpoint decrease. The unstable numerical value in the different runs is not considered for predicting the promoter of the increase/decrease endpoint [19, 56].

### Molecular docking

Molecular docking is a method commonly employed in drug discovery and development to identify protein–ligand binding configurations This approach involves the docking of a molecule with a specific macromolecule and then computing the binding free energy between the ligand and receptor[35]. The structure was sketched in ChemDraw 16.0, and the energy was minimised in Chem3D using the MM2 technique [57]. The crystallographic structure of Human cytochrome P450 CYP17A1 in complex with abiraterone was obtained from the Protein Data Bank (PDB: 3RUK) and used for molecular docking [58]. AutoDock Vina was employed for docking studies (Molecular Graphics Lab, CA, USA) [59]. The value of exhaustiveness was 8 and the dimensions of the grid box were 20.0, 20.0, and 20.0 Å in size. The findings and illustration were examined visually using Discovery Studio visualizer 2021.

## Results and discussion

### QSPR modelling for pIC_50_

Three types of outliers affect the model quality in QSPR/QSAR study. The first is the outliers in the dependent variable y, the second is the outliers in the direction of the independent variable X, and the third type of outliers indicates a different relationship between X and y. [60]. Here, based on several preliminary QSAR models, six compounds (compounds No. 31, 32, 36, 37, 67, and 80) identified as outliers, these molecules showed a large absolute error (> 3 s). These compounds fall in first type of outlier. The structure of these compounds is similar to the main body of the samples. So, they were removed from the data set before further data processing.

In this study, the balance of correlation approach was employed to generate QSAR models. A total of six QSAR models was generated utilizing two kinds of target functions i.e. TF_1_ (W_IIC_ = 0.0) and TF_2_ (W_IIC_ = 0.2). To obtain the preferable threshold value (T*) and the number of epochs (N*), the range of 1–10 for threshold and 1 to 50 for epoch were employed. In the case of TF_1_, the value of T* and N* were 1 and 10 for split 1; 1 and 3 for split 2; 1 and 7 for split 3, respectively. However, in the case of TF2, the value of optimum (T*, N*) for splits 1, 2, and 3 were (1, 10), (1, 10), and (1, 7), respectively.

The mathematical relationship for the developed QSAR model of pIC_50_ using TF_1_ and TF_2_ for three splits are displayed below:

The Monte Carlo optimization with target function TF_1_

15 $$\mathrm{Split }1\,\, {\mathrm{pIC}}_{50}=-8.4912\left(\pm 0.2835\right)+0.0978\left(\pm 0.0021\right)\times \mathrm{DCW}(1, 10)$$

16 $$\mathrm{Split }2\,\, {\mathrm{pIC}}_{50}=-16.266\left(\pm 0.2769\right)+0.1309\left(\pm 0.0017\right)\times \mathrm{DCW}(1, 3)$$

17 $$\mathrm{Split }3\,\, {\mathrm{pIC}}_{50}=-4.2842\left(\pm 0.2158\right)+0.0626\left(\pm 0.0015\right)\times \mathrm{DCW}(1, 7)$$

The Monte Carlo optimization with target function TF_2_

18 $$\mathrm{Split }1 \,\,{\mathrm{pIC}}_{50}=-3.1689\left(\pm 0.2140\right)+0.0272\left(\pm 0.0007\right)\times \mathrm{DCW}(1, 10)$$

19 $$\mathrm{Split }2\,\, {\mathrm{pIC}}_{50}=-9.6171\left(\pm 0.3420\right)+0.0758\left(\pm 0.0017\right)\times \mathrm{DCW}(1, 10)$$

20 $$\mathrm{Split }3\,\, {\mathrm{pIC}}_{50}=-7.0645\left(\pm 0.3206\right)+0.0482\left(\pm 0.0013\right)\times \mathrm{DCW}(1, 7)$$

The statistical results of designed QSAR models for three splits utilizing TF_1_ and TF_2_ are presented in Table 3. As can be seen, all developed QSAR models were acceptable statistically and agreed with the requirements of various validation criteria.

According to the results presented in Table 3, it was found that the models constructed using TF_2_ (with IIC) had better statistical results than the models constructed using TF_1_ (without IIC). The results of calibration and validation sets were better for the models constructed by using TF_2_, but the inferior quality of the model for the training sets was obtained. Hence, it can be expressed that the models designed with the IIC are more statistically considerable and robust for the present dataset. Based on validation metric study of QSPR/QSAR models by Ojha et al., the ${\overline{r} }_{m}^{2}$ value of models is used to judge the quality of the predictions by different models. The QSAR model developed by TF2 for split 3 was selected as a prominent model with highest ${\overline{r} }_{m}^{2}$ (${\overline{r} }_{m}^{2}$=0.615).

**Table 3:** The statistical characteristics of CORAL models for pIC_50_ generated with TF1 and TF2

Split	Target function	Set	n	R^2^	CCC	IIC	Q^2^	s	MAE	Y-rand	${cR}_{p}^{2}$	${\overline{r} }_{m}^{2}$	${\Delta r}_{m}^{2}$	F
1	TF1	TRN	29	0.813	0.897	0.551	0.779	0.234	0.189	0.033	0.797	–	–	117
		iTRN	20	0.813	0.866	0.607	0.773	0.275	0.229	0.050	0.788	–	–	78
		CAL	12	0.575	0.706	0.378	0.228	0.437	0.334	0.162	0.487	–	–	14
		VAL	19	0.679	0.765	0.402	0.549	0.364	0.259	–	–	0.510	0.279	36
	TF2	TRN	29	0.723	0.839	0.794	0.674	0.284	0.214	0.032	0.707	–	–	70
		iTRN	20	0.804	0.806	0.462	0.759	0.338	0.285	0.050	0.778	–	–	74
		CAL	12	0.688	0.802	0.828	0.552	0.313	0.267	0.064	0.655	–	–	22
		VAL	19	0.729	0.789	0.489	0.666	0.272	0.207	–	–	0.555	0.253	46
2	TF1	TRN	30	0.881	0.937	0.821	0.864	0.185	0.133	0.033	0.865	–	–	207
		iTRN	19	0.854	0.896	0.492	0.820	0.226	0.165	0.079	0.813	–	–	99
		CAL	11	0.502	0.653	0.279	0.029	0.546	0.378	0.087	0.456	–	–	9
		VAL	20	0.652	0.759	0.642	0.563	0.399	0.321	–	–	0.494	0.287	34
	TF2	TRN	30	0.781	0.877	0.773	0.740	0.251	0.175	0.027	0.767	–	–	100
		iTRN	19	0.805	0.831	0.865	0.752	0.278	0.216	0.049	0.780	–	–	70
		CAL	11	0.939	0.863	0.968	0.923	0.319	0.263	0.040	0.919	–	–	140
		VAL	20	0.775	0.826	0.611	0.713	0.334	0.269	–	–	0.613	0.218	62
3	TF1	TRN	30	0.736	0.848	0.751	0.674	0.288	0.204	0.043	0.714	–	–	78
		iTRN	18	0.754	0.853	0.856	0.704	0.277	0.231	0.077	0.714	–	–	49
		CAL	12	0.418	0.571	0.476	0.137	0.440	0.336	0.076	0.378	–	–	7
		VAL	20	0.651	0.798	0.651	0.457	0.302	0.231	–	–	0.518	0.190	34
	TF2	TRN	30	0.782	0.878	0.774	0.735	0.261	0.194	0.020	0.772	–	–	101
		iTRN	18	0.850	0.909	0.857	0.812	0.223	0.189	0.048	0.825	–	–	90
		CAL	12	0.777	0.807	0.881	0.697	0.300	0.246	0.100	0.725	–	–	35
		VAL	20	0.727	0.847	0.628	0.642	0.266	0.209	–	–	0.615	0.165	48

**Fig. 1:**
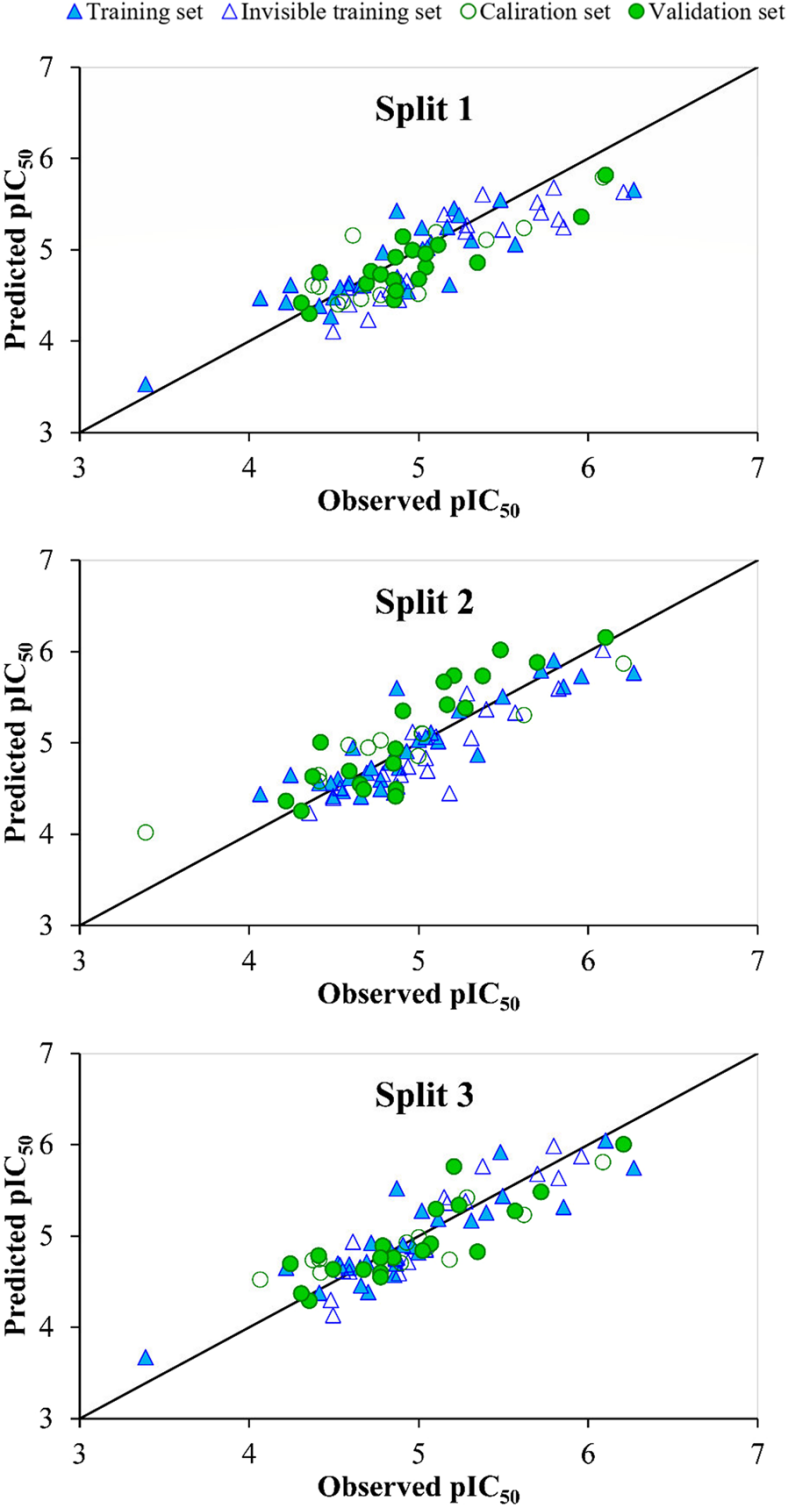
Observed pIC_50_ versus predicted pIC_50_ values for three CORAL models constructed based on TF_2_

The plot of observed pIC_50_ versus predicted pIC_50_ for three models designed with TF_2_ is displayed in Fig. 1. In the QSAR model generated by utilizing the Monte Carlo method, the outliers were introduced by the statistical defects. So, in the present QSAR model created by TF_2,_ the number of outliers was found six for all splits. Table 4 displays flavonols IDs, SMILES codes, and descriptor of correlation weights (DCWs) with their experimental and predicted pIC_50_.

**Table 4:** SMILES notation, the distribution of splits, DCWs, observed and predicted pIC_50_ of flavonols (+, −, #, and * show the componds located in the training, invisible training, calibration, and validation sets respectively)

No	SMILES	Split			_P_IC_50_	DCW(T, N)			Predicted pIC50		
		1	2	3		Split 1	Split 2	Split 3	Split 1	Split 2	Split 3
1	COc1ccc(cc1OC)C2=C(O)C(= O)c3ccccc3O2	+	#	+	3.39	246.56	179.87	222.70	3.53	4.02	3.67
2	COC1=C(Oc2ccccc2C1=O)c3ccc(OC)c(OC)c3	#	+	+	4.52	278.73	187.60	244.01	4.40	4.61	4.70
3	CCOC1=C(Oc2ccccc2C1=O)c3ccc(OC)c(OC)c3	*	*	−	4.86	297.66	191.93	245.04	4.92	4.94	4.75
4	CCCOC1=C(Oc2ccccc2C1=O)c3ccc(OC)c(OC)c3	#	#	+	5.00	282.96	190.91	246.53	4.52	4.86	4.82
5	CCCCOC1=C(Oc2ccccc2C1=O)c3ccc(OC)c(OC)c3	#	+	*	4.78	282.49	187.51	241.89	4.51	4.60	4.60
6	CCCCCOC1=C(Oc2ccccc2C1=O)c3ccc(OC)c(OC)c3	−	+	*	4.78	281.10	186.14	240.90	4.47	4.50	4.55
7	CCCCCCOC1=C(Oc2ccccc2C1=O)c3ccc(OC)c(OC)c3	*	−	+	4.85	280.45	185.60	241.36	4.45	4.46	4.57
8	CCCCCCCOC1=C(Oc2ccccc2C1=O)c3ccc(OC)c(OC)c3	+	+	#	4.06	281.26	185.40	240.36	4.47	4.44	4.52
9	COc1ccc(cc1OC)C2=C(OC(C)C)C(= O)c3ccccc3O2	+	+	−	4.48	273.82	187.00	235.69	4.27	4.56	4.30
10	CCC(C)OC1=C(Oc2ccccc2C1=O)c3ccc(OC)c(OC)c3	*	−	*	4.35	274.98	182.68	235.54	4.30	4.24	4.29
11	CCCC(C)OC1=C(Oc2ccccc2C1=O)c3ccc(OC)c(OC)c3	#	+	−	4.55	279.85	185.82	242.37	4.43	4.47	4.62
12	CCN(CC)CCCOC1=C(Oc2ccccc2C1=O)c3ccc(OC)c(OC)c3	+	+	*	4.24	286.50	188.17	243.94	4.62	4.65	4.70
13	CCN(CC)CCCCOC1=C(Oc2ccccc2C1=O)c3ccc(OC)c(OC)c3	+	*	+	4.22	279.62	184.40	243.02	4.43	4.37	4.65
14	CCN(CC)CCCCCOC1=C(Oc2ccccc2C1=O)c3ccc(OC)c(OC)c3	+	−	*	4.50	281.46	184.87	242.66	4.48	4.40	4.64
15	CCCN(CCC)CCCOC1=C(Oc2ccccc2C1=O)c3ccc(OC)c(OC)c3	−	#	+	4.59	285.56	192.48	243.07	4.59	4.98	4.66
16	CCCN(CCC)CCCCOC1=C(Oc2ccccc2C1=O)c3ccc(OC)c(OC)c3	−	*	−	4.59	278.69	188.71	242.15	4.40	4.69	4.61
17	CCCN(CCC)CCCCCOC1=C(Oc2ccccc2C1=O)c3ccc(OC)c(OC)c3	−	+	−	4.88	280.53	189.18	241.78	4.45	4.73	4.59
18	CCCCN(CCCC)CCCOC1=C(Oc2ccccc2C1=O)c3ccc(OC)c(OC)c3	−	+	+	5.85	309.77	200.92	256.83	5.25	5.62	5.32
19	CCCCN(CCCC)CCCCOC1=C(Oc2ccccc2C1=O)c3ccc(OC)c(OC)c3	+	−	*	5.57	302.90	197.16	255.91	5.06	5.33	5.27
20	CCCCN(CCCC)CCCCCOC1=C(Oc2ccccc2C1=O)c3ccc(OC)c(OC)c3	#	−	+	5.40	304.74	197.62	255.54	5.11	5.37	5.26
21	CCCCCN(CCCCC)CCCOC1=C(Oc2ccccc2C1=O)c3ccc(OC)c(OC)c3	#	#	#	5.62	309.50	196.79	255.03	5.24	5.31	5.23
22	CCCCCN(CCCCC)CCCCOC1=C(Oc2ccccc2C1=O)c3ccc(OC)c(OC)c3	*	+	+	5.11	302.63	193.03	254.11	5.05	5.02	5.19
23	CCCCCN(CCCCC)CCCCCOC1=C(Oc2ccccc2C1=O)c3ccc(OC)c(OC)c3	+	−	+	5.31	304.47	193.50	253.75	5.10	5.05	5.17
24	COc1ccc(cc1OC)C2=C(OCCCN3CCOCC3)C(= O)c4ccccc4O2	*	−	+	4.96	300.56	194.37	247.94	5.00	5.12	4.89
25	COc1ccc(cc1OC)C2=C(OCCCCN3CCOCC3)C(= O)c4ccccc4O2	*	−	−	5.04	293.68	190.60	247.02	4.81	4.84	4.85
26	COc1ccc(cc1OC)C2=C(OCCCCCN3CCOCC3)C(= O)c4ccccc4O2	*	+	*	5.35	295.52	191.07	246.66	4.86	4.87	4.83
27	COc1ccc(cc1OC)C2=C(OCCCCN3CCCCC3)C(= O)c4ccccc4O2	+	−	+	4.87	289.62	186.51	245.34	4.70	4.52	4.76
28	COc1ccc(cc1OC)C2=C(OCCCCCN3CCCCC3)C(= O)c4ccccc4O2	*	+	#	4.41	291.46	186.97	244.98	4.75	4.56	4.75
29	COc1ccc(cc1OC)C2=C(OCCCN3CCN(C)CC3)C(= O)c4ccccc4O2	+	*	+	5.48	320.82	206.21	269.28	5.55	6.02	5.92
30	COc1ccc(cc1OC)C2=C(OCCCCN3CCN(C)CC3)C(= O)c4ccccc4O2	*	+	−	5.96	313.95	202.44	268.36	5.36	5.73	5.87
33	COc1ccc(cc1OC)C2=C(OCCCCCCCN3CCN(C)CC3)C(= O)c4ccccc4O2	+	*	*	5.21	317.40	202.51	266.00	5.46	5.74	5.76
34	COc1ccc(cc1OC)C2=C(OCCCN3CCCC3)C(= O)c4ccccc4O2	−	*	−	5.70	319.85	204.41	264.33	5.52	5.88	5.68
35	COc1ccc(cc1OC)C2=C(OCCCCN3CCCC3)C(= O)c4ccccc4O2	−	−	−	5.82	312.98	200.64	263.41	5.33	5.60	5.64
38	COc1ccc(cc1OC)C2=C(OCCCCCCCN3CCCC3)C(= O)c4ccccc4O2	+	+	+	4.87	316.43	200.71	261.05	5.43	5.60	5.52
39	COc1ccc2C(= O)C(= C(Oc2c1)c3ccc(OC)c(OC)c3)O	−	#	+	4.70	272.50	192.11	237.49	4.24	4.95	4.39
40	COC1=C(Oc2cc(OC)ccc2C1=O)c3ccc(OC)c(OC)c3	−	+	+	4.83	284.69	189.92	247.43	4.57	4.78	4.87
41	CCOC1=C(Oc2cc(OC)ccc2C1=O)c3ccc(OC)c(OC)c3	+	+	*	5.07	303.63	194.26	248.46	5.08	5.11	4.92
42	CCCOC1=C(Oc2cc(OC)ccc2C1=O)c3ccc(OC)c(OC)c3	*	+	#	5.00	288.93	193.24	249.95	4.68	5.04	4.99
43	CCCCOC1=C(Oc2cc(OC)ccc2C1=O)c3ccc(OC)c(OC)c3	*	*	*	4.85	288.45	189.83	245.31	4.67	4.78	4.76
44	CCCCCOC1=C(Oc2cc(OC)ccc2C1=O)c3ccc(OC)c(OC)c3	*	+	+	4.69	287.07	188.46	244.33	4.63	4.67	4.72
45	CCCCCCOC1=C(Oc2cc(OC)ccc2C1=O)c3ccc(OC)c(OC)c3	#	*	#	4.37	286.42	187.93	244.78	4.61	4.63	4.74
46	CCCCCCCOC1=C(Oc2cc(OC)ccc2C1=O)c3ccc(OC)c(OC)c3	+	+	+	4.59	287.22	187.73	243.78	4.64	4.62	4.69
47	COc1ccc2C(= O)C(= C(Oc2c1)c3ccc(OC)c(OC)c3)OC(C)C	+	*	#	4.42	291.72	192.86	241.93	4.76	5.01	4.60
48	CCC(C)OC1=C(Oc2cc(OC)ccc2C1=O)c3ccc(OC)c(OC)c3	#	+	+	4.66	280.94	185.01	238.96	4.46	4.41	4.46
49	CCCC(C)OC1=C(Oc2cc(OC)ccc2C1=O)c3ccc(OC)c(OC)c3	#	#	*	4.41	285.82	188.15	245.80	4.60	4.65	4.79
50	CCCCN(CCCC)CCCOC1=C(Oc2cc(OC)ccc2C1=O)c3ccc(OC)c(OC)c3	−	+	*	5.72	315.74	203.25	260.25	5.41	5.79	5.48
51	CCCCN(CCCC)CCCCOC1=C(Oc2cc(OC)ccc2C1=O)c3ccc(OC)c(OC)c3	−	+	+	5.50	308.86	199.48	259.33	5.22	5.51	5.44
52	CCCCN(CCCC)CCCCCOC1=C(Oc2cc(OC)ccc2C1=O)c3ccc(OC)c(OC)c3	−	−	#	5.28	310.70	199.95	258.97	5.27	5.54	5.42
53	COc1ccc2C(= O)C(= C(Oc2c1)c3ccc(OC)c(OC)c3)OCCCN4CCOCC4	*	*	+	4.91	305.99	197.41	248.19	5.15	5.35	4.90
54	COc1ccc2C(= O)C(= C(Oc2c1)c3ccc(OC)c(OC)c3)OCCCCN4CCOCC4	*	+	−	5.04	299.12	193.64	247.27	4.96	5.07	4.86
55	COc1ccc2C(= O)C(= C(Oc2c1)c3ccc(OC)c(OC)c3)OCCCCCN4CCOCC4	+	#	*	5.02	300.96	194.11	246.91	5.01	5.10	4.84
56	COc1ccc2C(= O)C(= C(Oc2c1)c3ccc(OC)c(OC)c3)OCCCN4CCCCC4	#	+	−	4.61	306.52	192.08	248.94	5.16	4.95	4.94
57	COc1ccc2C(= O)C(= C(Oc2c1)c3ccc(OC)c(OC)c3)OCCCCN4CCCCC4	+	−	*	4.79	299.65	188.31	248.02	4.97	4.66	4.89
58	COc1ccc2C(= O)C(= C(Oc2c1)c3ccc(OC)c(OC)c3)OCCCCCN4CCCCC4	+	−	#	5.05	301.49	188.78	247.66	5.02	4.70	4.88
59	COc1ccc2C(= O)C(= C(Oc2c1)c3ccc(OC)c(OC)c3)OCCCN4CCN(C)CC4	*	*	+	6.10	330.85	208.01	271.96	5.82	6.16	6.05
60	COc1ccc2C(= O)C(= C(Oc2c1)c3ccc(OC)c(OC)c3)OCCCCN4CCN(C)CC4	−	#	*	6.21	323.98	204.24	271.04	5.63	5.87	6.00
61	COc1ccc2C(= O)C(= C(Oc2c1)c3ccc(OC)c(OC)c3)OCCCCCN4CCN(C)CC4	−	+	−	5.80	325.82	204.71	270.68	5.68	5.91	5.99
62	COc1ccc2C(= O)C(= C(Oc2c1)c3ccc(OC)c(OC)c3)OCCCN4CCCC4	#	−	#	6.09	329.88	206.21	267.01	5.79	6.02	5.81
63	COc1ccc2C(= O)C(= C(Oc2c1)c3ccc(OC)c(OC)c3)OCCCCN4CCCC4	−	*	−	5.38	323.01	202.44	266.09	5.61	5.73	5.77
64	COc1ccc2C(= O)C(= C(Oc2c1)c3ccc(OC)c(OC)c3)OCCCCCN4CCCC4	+	+	+	6.27	324.85	202.91	265.73	5.66	5.77	5.75
65	COc1cc(cc(OC)c1OC)C2=C(O)C(= O)c3ccccc3O2	−	+	−	4.49	267.80	185.10	232.23	4.11	4.42	4.13
66	COC1=C(Oc2ccccc2C1=O)c3cc(OC)c(OC)c(OC)c3	*	+	+	4.72	292.01	189.19	248.67	4.77	4.73	4.93
68	CCCOC1=C(Oc2ccccc2C1=O)c3cc(OC)c(OC)c(OC)c3	−	+	#	4.93	288.14	191.58	248.76	4.66	4.91	4.93
69	CCCCOC1=C(Oc2ccccc2C1=O)c3cc(OC)c(OC)c(OC)c3	+	−	#	4.89	287.66	188.18	244.11	4.65	4.65	4.71
70	CCCCCOC1=C(Oc2ccccc2C1=O)c3cc(OC)c(OC)c(OC)c3	+	*	+	4.65	286.28	186.81	243.13	4.61	4.55	4.66
71	CCCCCCOC1=C(Oc2ccccc2C1=O)c3cc(OC)c(OC)c(OC)c3	+	−	+	4.54	285.63	186.27	243.58	4.59	4.51	4.68
72	CCCCCCCOC1=C(Oc2ccccc2C1=O)c3cc(OC)c(OC)c(OC)c3	+	*	*	4.67	286.43	186.07	242.58	4.61	4.49	4.63
73	COc1cc(cc(OC)c1OC)C2=C(OC(C)C)C(= O)c3ccccc3O2	+	#	+	4.41	278.13	187.26	237.30	4.39	4.58	4.38
74	CCC(C)OC1=C(Oc2ccccc2C1=O)c3cc(OC)c(OC)c(OC)c3	*	*	*	4.31	279.29	182.94	237.15	4.42	4.25	4.37
75	CCCC(C)OC1=C(Oc2ccccc2C1=O)c3cc(OC)c(OC)c(OC)c3	*	*	+	4.87	284.16	186.08	243.98	4.55	4.49	4.70
76	CCN(CC)CCCCOC1=C(Oc2ccccc2C1=O)c3cc(OC)c(OC)c(OC)c3	−	*	+	4.86	284.80	185.07	245.25	4.57	4.42	4.76
77	CCN(CC)CCCCCOC1=C(Oc2ccccc2C1=O)c3cc(OC)c(OC)c(OC)c3	+	−	#	5.18	286.64	185.54	244.88	4.62	4.45	4.74
78	CCCN(CCC)CCCOC1=C(Oc2ccccc2C1=O)c3cc(OC)c(OC)c(OC)c3	*	#	*	4.77	290.74	193.15	245.29	4.73	5.03	4.76
79	CCCN(CCC)CCCCOC1=C(Oc2ccccc2C1=O)c3cc(OC)c(OC)c(OC)c3	+	−	−	4.94	283.86	189.38	244.37	4.54	4.74	4.72
81	CCCCN(CCCC)CCCOC1=C(Oc2ccccc2C1=O)c3cc(OC)c(OC)c(OC)c3	−	*	−	5.15	314.95	201.59	259.05	5.39	5.67	5.43
82	CCCCN(CCCC)CCCCOC1=C(Oc2ccccc2C1=O)c3cc(OC)c(OC)c(OC)c3	−	*	−	5.28	308.07	197.83	258.13	5.20	5.38	5.38
83	CCCCN(CCCC)CCCCCOC1=C(Oc2ccccc2C1=O)c3cc(OC)c(OC)c(OC)c3	+	*	−	5.17	309.91	198.29	257.77	5.25	5.42	5.36
84	CCCCCN(CCCCC)CCCOC1=C(Oc2ccccc2C1=O)c3cc(OC)c(OC)c(OC)c3	+	+	*	5.24	314.68	197.46	257.26	5.38	5.36	5.34
85	CCCCCN(CCCCC)CCCCOC1=C(Oc2ccccc2C1=O)c3cc(OC)c(OC)c(OC)c3	#	−	*	5.10	307.80	193.70	256.34	5.19	5.07	5.29
86	CCCCCN(CCCCC)CCCCCOC1=C(Oc2ccccc2C1=O)c3cc(OC)c(OC)c(OC)c3	+	#	+	5.02	309.64	194.16	255.97	5.24	5.11	5.28

### Interpretation of the QSAR model

The mechanistic interpretation of a QSAR model is the fifth principle of OECD. The mechanistic interpretation of the QSAR model provides a correlation and a relationship between the chemical structure of the compounds and their property/activity. It also enunciates the molecular features which are responsible for the increase/decrease of endpoints that can be computed from QSAR models. Information on the mechanistic interpretation of flavonols as a promoter of pIC_50_ increase/decrease may aid in the design and development of new flavonol derivatives.

In CORAL, correlation weights (CWs) of structural attributes (SA_k_) are calculated in three or more runs and the mechanistic interpretation is achieved by analysis of CWs. If in all probes of the optimization, the numerical value of CW of structural attributes is found greater than zero, then these attributes are considered as a promoter of increase. Whereas, if the numerical value of CW of structural attributes is found smaller than zero, then these attributes are defined as the promoter of decrease [61, 62].

**Fig. 2:**
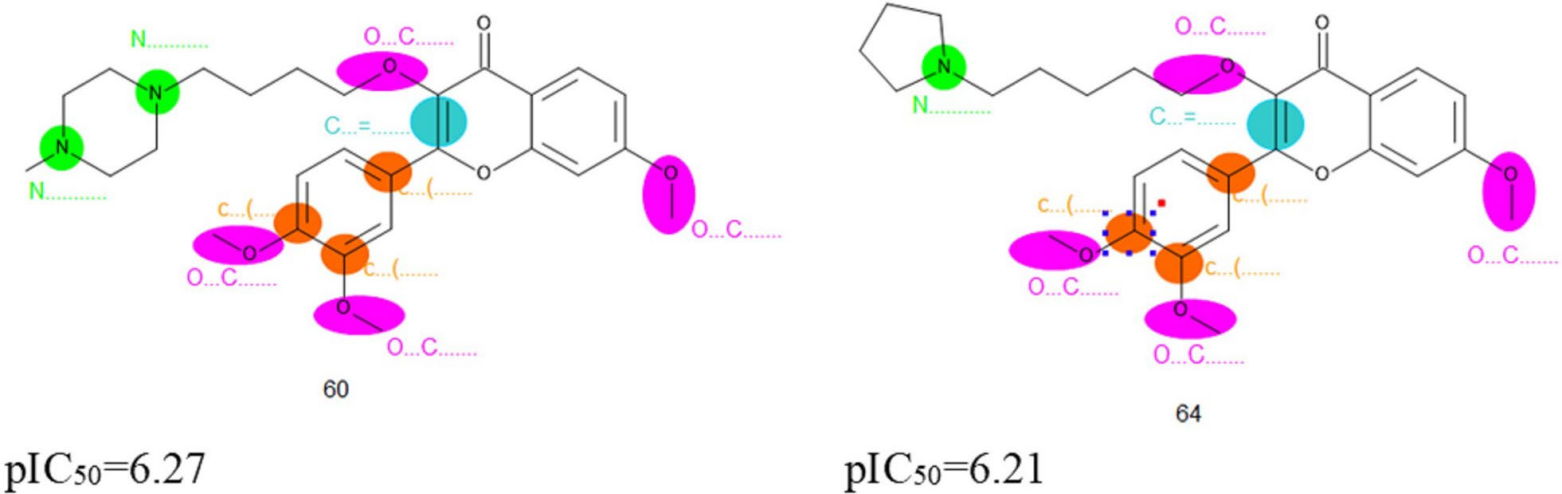
Good fingerprints obtained from Monte Carlo optimization method

The list of attributes and their correlation weights for three runs of all splits computed with TF_2_ is presented in Table 5. The most significant structural attributes as the promoter of pIC_50_ increase were distinguished and extracted. The structural attributes as promoters of increase of pIC_50_ were aliphatic carbon atom connected to double-bound (C…=…, aliphatic oxygen atom connected to aliphatic carbon (O…C…), branching on aromatic ring (c…(…), and aliphatic nitrogen (N…). The good fingerprints obtained from Monte Carlo optimization method are indicated in Fig. 2. These attributes for two compounds with the highest pIC_50_ are shown in Fig. 2 (compound no. 60 and 64).

**Table 5:** Important features interpretation for increasing of pIC_50_ values of three splits

No	Attributes	Split	CW (M_Fk_) in run 1	CW (M_Fk_) in run 2	CW (M_Fk_) in run 3	N_T_^a^	N_iT_^b^	N_C_^c^	Defect (M_Fk_)	Interpretation
1	C…=…	1	1.080	1.495	0.261	29	20	12	0.000	Aliphatic carbon atom connected to double-bound
		2	0.810	1.103	0.681	29	21	12	0.000	
		3	1.978	1.006	0.076	28	20	12	0.000	
2	O…C…	1	1.216	1.517	0.893	29	20	12	0.000	Aliphatic oxygen atom connected to aliphatic carbon
		2	0.014	0.254	0.380	29	21	12	0.000	
		3	0.150	0.019	1.054	28	20	12	0.000	
3	c…(…	1	0.329	0.003	0.560	29	20	12	0.000	Branching on ring
		2	0.271	0.054	0.161	29	21	12	0.000	
		3	0.263	0.073	0.644	28	20	12	0.000	
4	N…	1	2.197	3.253	3.130	19	10	6	0.0062	Aliphatic nitrogen
		2	0.050	0.207	0.170	18	11	7	0.0015	
		3	0.728	2.431	2.322	16	15	6	0.0032	
		2	-0.718	-0.708	− 0.854	22	16	10	0.0023	
		3	-0.285	-0.784	− 1.012	22	16	9	0.0012	

^a^No. of attributes in the training set

^b^No. of attributes in the invisible training set

^c^No. of attributes in the calibration set

A series of natural flavonols with unknown pIC_50_ was selected and their pIC_50_ was calculated from the QSAR models of best split (split 3). Names, chemical structure and corresponding predicted pIC_50_ of selected natural flavonol derivatives with pIC_50_ more than 4, are depicted in Table 6. These compounds were also considered for molecular docking studies.

**Table 6:** The chemical structure of some natural flavonols with predicted pIC_50_ using leading model (split 3), docking scores (Kcal mol^−1^) and amino acid interacted with 3RUK

Structure	pIC_50_ (prd)	Free binding energy (kcal/mol)	Amio acid interacted
Abiraterone	–	− 10.3	Ala113, Gly301, Arg239, Asn202, Ile206, Glu305, Ala302, Val482, Val483, Ile371, Ile205, Phe114, Thr306, Cys442, Val366, Ala367, Leu209, Tyr201
Compound no. 60 with highest Activity	6.03	− 8.1	Ala113, Gly301, Asp298, Gly444, Val483, Val366, Ala302, Ala367, Ile299, Ile443, Ile371, Glu305, Phe435, Cys442, Gly303, Ile371, Pro434, Leu447, Ile112, Phe114, Thr306
Azaleatin	4.36	− 8.1	Ile205, Val482, Asp298, Ala302, Ala113, Asn202, Gly297
Gossypetin	4.59	− 8.5	Asn202, Arg239 Ile205, Ala105, Ala302, Ala113
Isorhamnetin	4.68	− 8.0	Ala113, Gly301, Asp298, Gly297, Arg239, Val236, Ala105, Ile205, Tyr201, Asn202, Ile206, Glu305, Ala302, Val482, Val483, Ile371
Myricetin	4.21	− 8.2	Ala113, Gly301, Asp298, Gly297, Arg239, Phe114, Phe300, Ile205, Tyr201, Asn202, Ile206, Glu305, Ala302, Val482, Val483, Ile371
Pachypodol	4.35	− 7.9	Ala113, Ala105, Gly301, Arg239, Ile205, Asn202, Ile206, Glu305, Ala302, Val482, Val483, Ile371, Val236, Thr306
Quercetin	4.25	− 8.4	Ala113, Ala105, Gly301, Arg239, Ile205, Asn202, Ile206, Glu305, Ala302, Val482, Val483, Il371, Val236, Thr306, Phe114, Tyr201
Rhamnazin	4.66	-8.3	Ala113, Ala105, Gly301, Arg239, Ile205, Asn202, Ile206, Glu305, Ala302, Val482, Val483, Ile371, Val236, Tyr201, Asp298, Gly297, Ala367, Ala105, Val366
Rhamnetin	4.45	− 8.2	Ala113, Gly301, Arg239, Ile205, Asn202, Ile206, Ala302, Val482, Val483, Ile371, Asp298, Gly297, Ala367, Tyr201

### Molecular docking studies

**Fig. 3:**
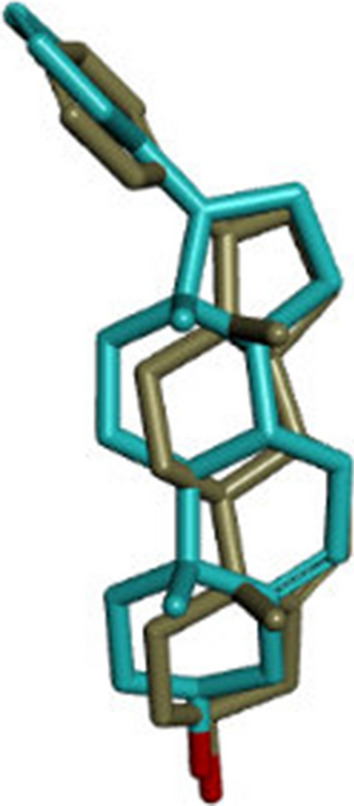
Superposition of the abiraterone output docked ligand (blue) and the co-crystallized ligand (green) of 3RUKA

**Fig. 4:**
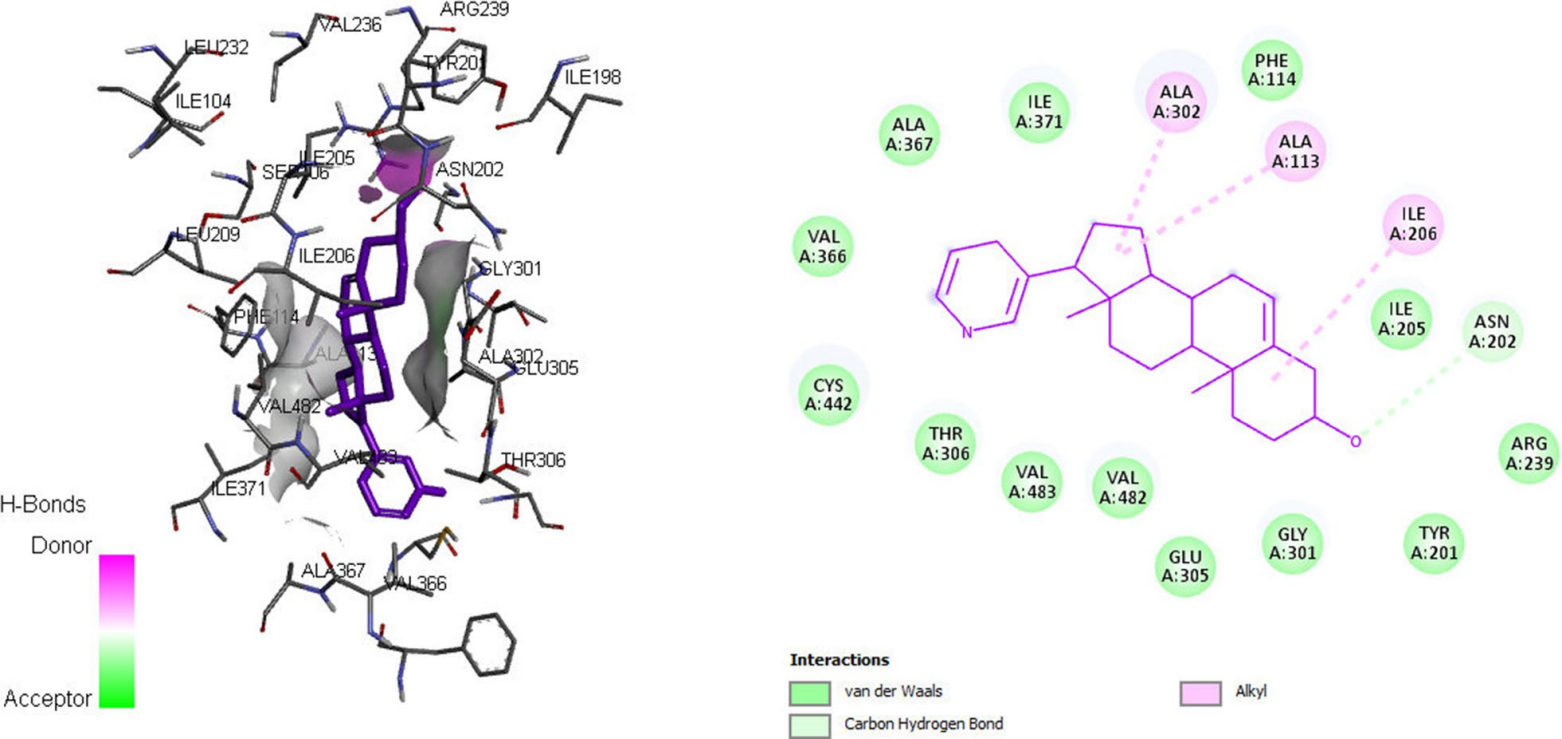
3D docking mode and 2D schematic interaction diagram for the best pose of abiraterone redocked into 3RUK crystal structure

The docking for abiraterone was performed into the active site of Human Cytochrome P450 CYP17A1 (PDB: 3RUK) to validate the binding energy of ligand–protein interactions. The validation results showed a binding energy of − 10.3 kcal/mol for abiraterone and a root-mean-square deviation (RMSD) value 1.172 Å (Fig. 3). The active pocket consisted of amino acid residues such as Val366, Val483, Val482, Ala367, Glu305, Gly301, Leu209, Asn220, Tyr201, Ile206, Ile205, Arg239, Phe114, ala302, Ile371, Ala113, Thr306, and Cys442, which play fundamental roles by hydrophobic interactions and forming H-bond (Fig. 4).

**Fig. 5:**
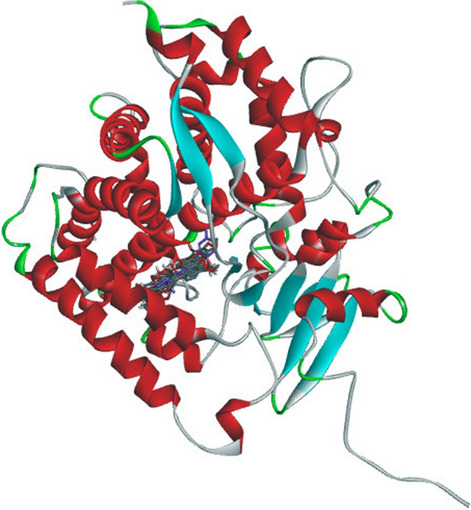
Superimposed poses of docked molecules and the co-crystallized abiraterone (violet) into the active site of 3RUKA

**Fig. 6:**
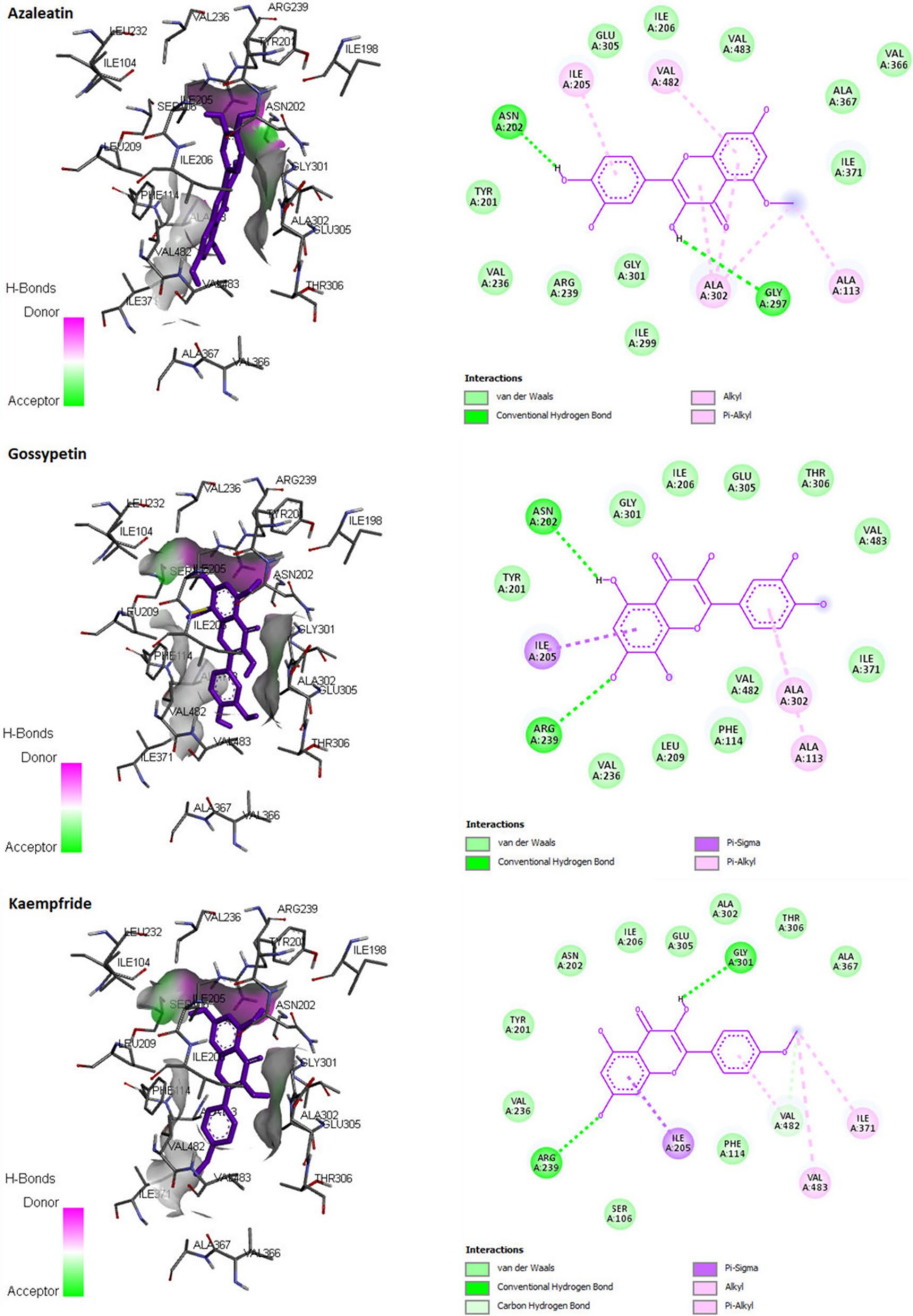
3D docking mode and 2D schematic interaction diagram for the best pose of some natural flavonols against 3RUK crystal structure (for interpretation of the references to color in this figure legend, the reader is referred to the web version of this article)

In addition, the docking studies for eight natural flavonols with predicted pIC_50_ more than 4.0 based on the best model (split 3), were conducted along with compound number 60, which has high experimental activity. Natural flavonols azaleatin, gossypetin, isorhamnetin, myricetin, pachypodol, quercetin, rhamnazin, and rhamnetin exhibited binding energies of − 8.1, − 8.5, − 8.0, − 8.2, − 7.9, − 8.4, − 8.3, and − 8.2 kcal/mol, respectively (Table 6). The docking outcomes matched the calculated pIC_50_ of flavonols. The superimposition image of the optimum binding pose for each suggested flavonol is displayed in Fig. 5. Figure 6 shows the 3D docking mode and 2D schematic depiction of interactions for some natural flavonols and the active ligand. The oxygen atom was involved in hydrogen bond interactions with the active site amino acid residues, and so the oxygen of flavonols was particularly significant for the anti-prostate cancer effect of flavonols. The positive contribution of oxygen atom on pIC_50_ of flavonol derivatives was seen in the mechanistic interpretation of the above-mentioned QSAR models. So, the present QSAR models are acceptable for a wide range of flavonols derivatives.

## Conclusion

In the present study, a reliable QSAR model was described to predict the anti-prostate cancer activities of 81 flavonol derivatives using the Monte Carlo optimization technique of CORAL software. To date, the QSAR models to predict the pIC_50_ of this dataset were not previously reported. Six QSAR models were constructed utilizing the balance of correlation method with two target functions TF_1_ (WIIC = 0.0) and TF_2_ (WIIC = 0.2). The IIC was employed to improve the reliability and robustness of the models. The QSAR models developed by using TF2 were found better than the models developed by TF_1_. The predictability and robustness of designed models were evaluated by the various statistical parameters such as R^2^, Q^2^, IIC, CCC, MAE, s, $\overline{{r }_{m}^{2}}$, Δ${r}_{m}^{2}$, ${C}_{{R}_{p}^{2}}$, F and Y-test. Based on ‘statistical defect’, d(A) for a SMILES attribute, the AD was also analysed and the outliers were extracted. The structural attributes as promoters of increase/decrease of pIC_50_ were identified and used to predict the pIC_50_ of natural flavonols. The mechanistic interpretation was also confirmed by molecular docking of natural flavonols into the active site of Human Cytochrome P450 CYP17A1 (PDB: 3RUK).

## Supplementary Information

**Additional file 1: Table S1.** Chemical structures of flavonol derivatives and IC50 values against PC-3 prostate cancer cells.

## Data Availability

The datasets used and/or analyzed during the current study are available from the corresponding author on reasonable request.
