# Supplementary material for: In-silico activity prediction and docking studies of some flavonol derivatives as anti-prostate cancer agents based on Monte Carlo optimization
Source: BMC Chem. 2023 Jul 26;17(1):87. doi: 10.1186/s13065-023-00999-y (PMC10373329; doi:10.1186/s13065-023-00999-y)
Supplement: Supplementary file 1 — Additional file 1: Table S1. Chemical structures of flavonol derivatives and IC50 values against PC-3 prostate cancer cells. [file 13065_2023_999_MOESM1_ESM.docx]

**Table S1**. Chemical structures of flavonol derivatives and IC_50_ values against PC-3 prostate cancer cells.

|  |  | Group 1   | | |
| --- | --- | --- | --- | --- |
| Ref. | pIC_50_ | IC_50_ (μM) | R | No. |
| [1] | 3.39 | 409.0 | H | 1 |
| [1] | 4.52 | 30.0 | Methyl | 2 |
| [1] | 4.86 | 13.7 | Ethyl | 3 |
| [1] | 5.00 | 10.1 | Propyl | 4 |
| [1] | 4.77 | 16.8 | Butyl | 5 |
| [1] | 4.77 | 16.8 | Pentyl | 6 |
| [1] | 4.85 | 14.0 | Hexyl | 7 |
| [1] | 4.06 | 86.3 | Heptyl | 8 |
| [1] | 4.48 | 33.0 | Isopropyl | 9 |
| [1] | 4.35 | 44.2 | Sec-Butyl | 10 |
| [1] | 4.55 | 28.1 | Pentan-2-yl | 11 |
| [1] | 4.24 | 57.2 | (N,N-Diethylamino)propyl | 12 |
| [1] | 4.22 | 60.7 | (N,N-Diethylamino)butyl | 13 |
| [1] | 4.49 | 32.0 | (N,N-Diethylamino)pentyl | 14 |
| [1] | 4.58 | 26.0 | (N,N-Dipropylamino)propyl | 15 |
| [1] | 4.59 | 25.7 | (N,N-Dipropylamino)butyl | 16 |
| [1] | 4.88 | 13.1 | (N,N-Dipropylamino)pentyl | 17 |
| [1] | 5.85 | 1.4 | (N,N-Dibutylamino)propyl | 18 |
| [1] | 5.57 | 2.7 | (N,N-Dibutylamino)butyl | 19 |
| [1] | 5.40 | 4.0 | (N,N-Dibutylamino)pentyl | 20 |
| [1] | 5.62 | 2.4 | (N,N-Dipentylamino)propyl | 21 |
| [1] | 5.11 | 7.7 | (N,N-Dipentylamino)butyl | 22 |
| [1] | 5.31 | 4.9 | (N,N-Dipentylamino)pentyl | 23 |
| [1] | 4.96 | 10.9 | Morpholinopropyl | 24 |
| [1] | 5.04 | 9.1 | Morpholinobutyl | 25 |
| [1] | 5.35 | 4.5 | Morpholinopentyl | 26 |
| [1] | 4.87 | 13.4 | Piperidinobutyl | 27 |
| [1] | 4.41 | 38.6 | Piperidinopentyl | 28 |
| [1] | 5.48 | 3.3 | (4-Methylpiperazin-1-yl)propyl | 29 |
| [1] | 5.96 | 1.1 | (4-Methylpiperazin-1-yl)butyl | 30 |
| [1] | 6.30 | 0.5 | (4-Methylpiperazin-1-yl)pentyl | 31 |
| [1] | 4.70 | 19.8 | (4-Methylpiperazin-1-yl)hexyl | 32 |
| [1] | 5.21 | 6.2 | (4-Methylpiperazin-1-yl)heptyl | 33 |
| [1] | 5.70 | 2.0 | Pyrrolidinopropyl | 34 |
| [1] | 5.82 | 1.5 | Pyrrolidinobutyl | 35 |
| [1] | 6.15 | 0.7 | Pyrrolidinopentyl | 36 |
| [1] | 4.76 | 17.4 | Pyrrolidinohexyl | 37 |
| [1] | 4.87 | 13.5 | Pyrrolidinoheptyl | 38 |
|  |  | Group 2   | | |
| [1] | 4.70 | 19.9 | H | 39 |
| [1] | 4.83 | 14.9 | Methyl | 40 |
| [1] | 5.07 | 8.5 | Ethyl | 41 |
| [1] | 5.00 | 10.0 | Propyl | 42 |
| [1] | 4.85 | 14.1 | Butyl | 43 |
| [1] | 4.69 | 20.3 | Pentyl | 44 |
| [1] | 4.37 | 42.3 | Hexyl | 45 |
| [1] | 4.59 | 25.7 | Heptyl | 46 |
| [1] | 4.42 | 38.0 | Isopropyl | 47 |
| [1] | 4.66 | 22.0 | Sec-butyl | 48 |
| [1] | 4.41 | 39.0 | Pentan-2-yl | 49 |
| [1] | 5.72 | 1.9 | (N,N-Dibutylamino)propyl | 50 |
| [1] | 5.49 | 3.2 | (N,N-Dibutylamino)butyl | 51 |
| [1] | 5.28 | 5.2 | (N,N-Dibutylamino)pentyl | 52 |
| [1] | 4.91 | 12.4 | Morpholinopropyl | 53 |
| [1] | 5.04 | 9.1 | Morpholinobutyl | 54 |
| [1] | 5.02 | 9.5 | Morpholinopentyl | 55 |
| [1] | 4.61 | 24.5 | Piperidinopropyl | 56 |
| [1] | 4.79 | 16.3 | Piperidinobutyl | 57 |
| [1] | 5.05 | 8.9 | Piperidinopentyl | 58 |
| [1] | 6.10 | 0.79 | (4-Methylpiperazin-1-yl)propyl | 59 |
| [1] | 6.21 | 0.62 | (4-Methylpiperazin-1-yl)butyl | 60 |
| [1] | 5.80 | 1.6 | (4-Methylpiperazin-1-yl)pentyl | 61 |
| [1] | 6.09 | 0.82 | Pyrrolidinopropyl | 62 |
| [1] | 5.38 | 4.2 | Pyrrolidinobutyl | 63 |
| [1] | 6.27 | 0.54 | Pyrrolidinopentyl | 64 |
|  |  | Group 3   | | |
| [2] | 4.49 | 32.1 | H | 65 |
| [2] | 4.72 | 19.1 | Methyl | 66 |
| [2] | 6.05 | 0.90 | Ethyl | 67 |
| [2] | 4.93 | 11.8 | Propyl | 68 |
| [2] | 4.89 | 12.8 | Butyl | 69 |
| [2] | 4.65 | 22.2 | Pentyl | 70 |
| [2] | 4.53 | 29.2 | Hexyl | 71 |
| [2] | 4.67 | 21.2 | Isopropyl | 72 |
| [2] | 4.41 | 38.6 | Sec-butyl | 73 |
| [2] | 4.30 | 49.3 | Pentan-2-yl | 74 |
| [2] | 4.87 | 13.6 | (N,N-diethylamino)propyl | 75 |
| [2] | 4.86 | 13.7 | (N,N-diethylamino)butyl | 76 |
| [2] | 5.18 | 6.6 | (N,N-diethylamino)pentyl | 77 |
| [2] | 4.77 | 16.8 | (N,N-dipropylamino)propyl | 78 |
| [2] | 4.94 | 11.6 | (N,N-dipropylamino)butyl | 79 |
| [2] | 5.85 | 2.6 | (N,N-dipropylamino)pentyl | 80 |
| [2] | 5.15 | 7.1 | (N,N-dibutylamino)propyl | 81 |
| [2] | 5.28 | 5.3 | (N,N-dibutylamino)butyl | 82 |
| [2] | 5.17 | 6.8 | (N,N-dibutylamino)pentyl | 83 |
| [2] | 5.24 | 5.8 | (N,N-dipentylamino)propyl | 84 |
| [2] | 5.10 | 7.9 | (N,N-dipentylamino)butyl | 85 |
| [2] | 5.02 | 9.6 | (N,N-dipentylamino)pentyl | 86 |

[1] X. Li, C. Zhang, S. Guo, P. Rajaram, M. Lee, G. Chen, R. Fong, A. Gonzalez, Q. Zhang, S. Zheng, Structure-activity relationship and pharmacokinetic studies of 3-O-substitutedflavonols as anti-prostate cancer agents, European journal of medicinal chemistry 157 (2018) 978-993.

[2] X. Li, M. Lee, G. Chen, Q. Zhang, S. Zheng, G. Wang, Q.-H. Chen, 3-O-Substituted-3′, 4′, 5′-trimethoxyflavonols: Synthesis and cell-based evaluation as anti-prostate cancer agents, Bioorganic & medicinal chemistry 25(17) (2017) 4768-4777.
